# Motion dazzle and the effects of target patterning on capture success

**DOI:** 10.1186/s12862-014-0201-4

**Published:** 2014-09-13

**Authors:** Anna E Hughes, Jolyon Troscianko, Martin Stevens

**Affiliations:** Department of Physiology, Development, and Neuroscience, University of Cambridge, Downing Street, Cambridge, CB2 3EG UK; Centre for Ecology and Conservation, University of Exeter, Penryn Campus, Penryn, Cornwall, TR10 9FE UK

**Keywords:** Motion dazzle, Vision, Animal coloration

## Abstract

**Background:**

Stripes and other high contrast patterns found on animals have been hypothesised to cause “motion dazzle”, a type of defensive coloration that operates when in motion, causing predators to misjudge the speed and direction of object movement. Several recent studies have found some support for this idea, but little is currently understood about the mechanisms underlying this effect. Using humans as model ‘predators’ in a touch screen experiment we investigated further the effectiveness of striped targets in preventing capture, and considered how stripes compare to other types of patterning in order to understand what aspects of target patterning are important in making a target difficult to capture.

**Results:**

We find that striped targets are among the most difficult to capture, but that other patterning types are also highly effective at preventing capture in this task. Several target types, including background sampled targets and targets with a ‘spot’ on were significantly easier to capture than striped targets. We also show differences in capture attempt rates between different target types, but we find no differences in learning rates between target types.

**Conclusions:**

We conclude that striped targets are effective in preventing capture, but are not uniquely difficult to catch, with luminance matched grey targets also showing a similar capture rate. We show that key factors in making capture easier are a lack of average background luminance matching and having trackable ‘features’ on the target body. We also find that striped patterns are attempted relatively quickly, despite being difficult to catch. We discuss these findings in relation to the motion dazzle hypothesis and how capture rates may be affected more generally by pattern type.

## Background

Camouflage can be defined as “strategies involved in concealment, including prevention of detection and recognition” [1], and offers an important anti-predator defence for many species. One common type of camouflage is crypsis, which helps to prevent initial detection of an object through a variety of mechanisms, from simple background matching through to disruptive camouflage and self-shadow concealment [2]. Many studies of camouflage have considered the case in which a prey item is stationary on its background (e.g. [3-9]). However, most animals cannot remain still indefinitely and often need to move to find food and mates, becoming especially vulnerable to detection and attack during these periods. It is therefore important to ask whether some types of patterning can provide protection from predator attack when in motion.

Recent research into this area has focused specifically on concept of ‘motion dazzle’, where high contrast stripe and zigzag markings are hypothesised to ‘dazzle’ an approaching predator, making it difficult for them to judge the speed and direction of the animal’s movement [1]. This concept was proposed by Thayer and others and was applied in World War I and II to the painting of some Allied ships [10]. It was theorised that using striking geometric patterns would make it difficult to target a moving ship accurately by making the trajectory or speed of the vessel difficult to estimate, although evidence obtained at the time was inconclusive about the effectiveness of the dazzle markings [11]. From an ecological perspective, several studies have suggested that the high contrast markings seen on animals such as zebras and some snakes may create visual illusions, distorting speed or direction perception in some manner [12-16].

The first experimental test of the motion dazzle hypothesis was conducted by Stevens and colleagues, who ran human artificial ‘prey’ capture experiments where subjects were required to attempt to capture moving targets in a computer game. They found that some targets with highly conspicuous patterns, such as bands and zigzags, were more difficult to capture than uniformly coloured, luminance matched conspicuous stimuli [17]. Scott-Samuel and colleagues investigated the potential mechanisms behind dazzle camouflage, in a task where human subjects were asked to judge which of two targets appeared to be moving faster, in order to determine the point at which they were subjectively perceived to be travelling at the same speed. They found that dazzle patterning could affect speed perception in humans, with high contrast patterns causing a reduction in perceived speed [18]. However, they did not find a significant effect for striped stimuli, with only some high contrast 2D patterns (such as zigzags and checks) causing perceived speed reductions.

However, not all research has supported the motion dazzle hypothesis. One study combined a number of approaches, asking human participants to both attempt to capture moving stimuli and also to make perceptual judgements of speed in separate experiments [19]. This challenged the finding that high contrast patterning causes a reduction in perceived speed, suggesting that striped targets are in fact perceived as moving faster than non-patterned targets and also arguing that striped targets are no more difficult, or perhaps sometimes easier, than non-patterned targets to capture [19]. A further study used cuttlefish (*Sepia officinalis*) as a means to test whether dazzle patterning is observed in a more natural system [20]. As cuttlefish are able to change their body patterns rapidly in response to their surroundings, the researchers asked whether they would be more likely to use low contrast mottled patterns or high contrast ‘dazzle’ patterns when in motion. They found that cuttlefish reduced the amount of high contrast patterns they displayed when in motion, and concluded that they did not use motion dazzle patterning for the purpose of motion camouflage. Similarly, several human studies have found that low contrast striped targets are more difficult to capture than high contrast ones [17,21]. There is therefore still much debate as to the efficacy of motion dazzle, and the mechanisms that underlie it.

Some data have suggested that other patterning types may be effective in preventing capture. Background matching stimuli (plain grey luminance-matched) have also been shown to be effective at reducing capture rates compared to other target types tested, including ‘motion dazzle’ type stimuli [17], suggesting that cryptic, camouflage markings may offer good protection when in motion as well as when stationary. However, other results have suggested that background matching may not be as effective as ‘motion dazzle’ patterning; in one study, background matching camouflaged stimuli were found to be harder to detect than high contrast striped stimuli when they were stationary, but were caught more often than the same stimuli when they were moving [21]. It is therefore also unclear to what extent cryptic camouflage strategies offer protection against capture in motion.

It is not fully understood what aspects of a target’s pattern are most critical for making capture difficult, and by what mechanism motion dazzle could work. One phenomenon which may be of critical importance is “aperture problem”, which occurs because the cells involved in the early stages of the human visual system in processing motion information have small, local receptive fields. The motion of a line through these receptive fields is ambiguous for motion parallel to the line itself, with only movement perpendicular to the line being detectable [22]. Unambiguous ‘features’ in the image such as corners, contour end points or points of high curvature do not suffer from the aperture problem and therefore may be used to estimate the true speed and direction of motion [23-28]. These features may be detected by end stopped cells in primary visual cortex in primates [29], which allows segmentation of the visual scene [30] and then tracking of the features over time [31,32]. It has been hypothesised that the terminator signals may then propagate along the ambiguous segments of contours, constraining the velocity and direction signals, allowing recovery of object motion [31]. It may therefore be the case that targets without obvious features may be most difficult to catch. In addition, there may be some feature types that are better at preventing the aperture effect and allowing the true motion to be determined than others. Recent research involving modelling potential motion detection mechanisms in vision showed that the stripes on zebra create erroneous information about direction of movement, much more so than unpatterned horses [16]. The researchers hypothesised that these erroneous motion signals may be caused both by the aperture effect and the wagon-wheel effect, where motion is perceived to be inverted by spatiotemporal aliasing (although there is still debate as to whether this effect can happen under natural viewing conditions [16]).

Recent research has shown that observers learn to detect types of static camouflage at different rates [33]. Learning effects have not previously been investigated in the context of moving stimuli, but if there are differences in learning rates between different patterns when targets are in motion, this may suggest that there are qualitatively or quantitatively different perceptual or cognitive processes involved in the capture process for different stimuli, and thus may help to explain the differences between pattern types.

While there have now been several studies considering both the hypothesis of motion dazzle and more generally how patterning affects perceptual and behavioural judgements when in motion (reviewed in Table 1), there is still debate as to which strategies are optimal and what aspects of a target’s pattern are important in determining capture difficulty. In this study, we use human prey capture experiments similar to those conducted by Stevens and colleagues [17,21] to investigate these questions. We compare putative ‘motion dazzle’ transverse striped targets, different types of cryptic stimuli (uniform luminance matched grey and background matching targets) and highly conspicuous white targets to determine how these different patterning types compare in difficulty of capture. We predict from previous work [17,21] that striped targets will be among the most difficult to capture, with white targets and background matching targets being more easily caught. As the existence of motion dazzle is still contentious, attempting to replicate these previous findings is important. We also extend previous research to consider the effect of several types of targets patterned with conspicuous white markings (a white edged target, and a target with a central white spot), predicting that these will increase capture success by providing ‘features’ for the observers to track. Furthermore, we tested whether participants improved their capture success at different rates for different patterning types to consider whether there are differences in learning for different target types and whether this can explain the patterns of results seen.

**Table 1:** **Review of previous ‘motion dazzle’ studies**

**Paper**	**Methodology**	**Conclusions**
Stevens et al. (2008) [17]	Human ‘prey capture’ experiments using a computer game. Self paced responses, unpredictable trajectory.	Targets with highly conspicuous stripe/zigzag patterns & uniformly camouflaged (grey) targets similarly difficult to catch and caught less than some other pattern types.
Zylinski et al. (2009) [20]	Tested whether cuttlefish were more likely to use low or high contrast (‘dazzle’) patterns when in motion.	Cuttlefish reduced the amount of high contrast patterns they displayed when in motion.
Scott-Samuel et al. (2011) [18]	Humans asked to judge which of two patterns appeared to be moving more quickly. Predictable trajectory.	Targets with zigazag/check patterns perceived to be moving more slowly than unpatterned (white Gaussian) targets. No effect for striped patterns.
Stevens et al. (2011) [21]	Human ‘prey capture’ experiments using a touch screen computer game. Self paced responses, unpredictable trajectory.	Striped moving targets caught less often than camouflaged (background matching) targets (despite being caught more often when stationary).
Santer (2013) [34]	Tested response of locust neurons involved in escape responses to motion dazzle stimuli.	High contrast motion dazzle stimuli caused a weaker response in these neurons than uniformly dark stimuli. However, uniformly bright stimuli produce an even weaker response.
Von Helversen et al. (2013) [19]	Humans asked to attempt to capture moving target using a joystick. Target moving on predictable trajectory and disappeared before capture attempt made. Also made perceptual judgements about which of two patterns appeared to be moving more quickly.	Striped targets no more difficult or easier than uniform black targets to capture. Striped targets perceived as moving faster than uniform black targets.
How & Zanker (2014)[16]	Modelling potential motion detection mechanism in human vision and the motion signals that zebras would produce in this model.	Stripes on zebras produce more erroneous information about direction of movement than unpatterned horses.

Previous experiments in this area have often allowed participants to make capture attempts at their own pace [17,18,21]. For example, in several previous capture studies [17,21], targets were presented for a fixed period of time (e.g. one minute) and participants were instructed to try to catch the target as many times as possible in that interval. In the current study, targets were only present on screen for a brief period in each trial, and therefore participants needed to make fast responses to have a chance of capturing the target. This design was chosen to allow us to standardise how participants had to approach the task, and may also correspond to natural situations where animals are only visible for short periods of time; e.g. if they are moving between two different patches of occluding vegetation. We then investigated whether there were differences in capture attempt times for different target types, and how this might relate to the detectability of targets and how confident subjects felt in their judgements, as confidence judgements and reaction times are thought to be inversely related [35-37]

## Methods

**Figure 1:**
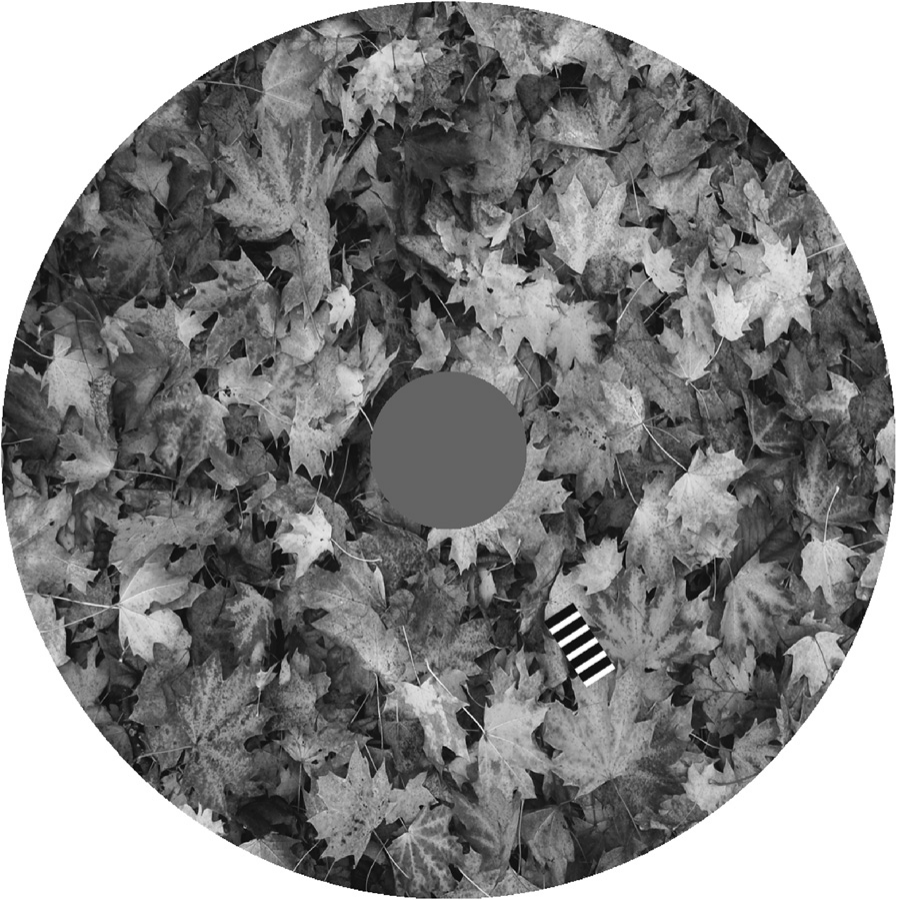
**An example screen shot showing the general set up of the experiment.**

The experiment was a computer ‘game’ created in Multimedia Fusion 2 (Clickteam 1996–2011) and played on a touch screen monitor (Elo 1515 L; Tyco Electronics, Shanghai, China, 1280 × 1024 pixels, or 42.85 × 34.28 degrees subtended on the viewer’s eye) by human subjects. The achromatic target (90×40 pixels large, 3 × 1.33 degrees or approximately 24 × 11 mm) started behind an occluding circle (diameter 179 pixels, 5.99 degrees) in the centre of the screen, luminance matched to the average background luminance. The target then moved out in a random direction at a speed of 20.8 cm/s (approximately 26.7 degrees of visual angle per second) through a circular arena (diameter 1024 pixels, 34.28 degrees) before disappearing. The subjects’ task was to make a capture attempt before the target left the circular arena. The target did not change trajectory once it had started moving. After the subject touched the screen, a cross appeared on the screen in the position they had clicked. The colour of this cross indicated whether they had hit or missed the target (green or red, respectively). The computer program recorded the position of the capture attempt, the position of the target at the time of the capture attempt, the time of the capture attempt and whether the subject had hit or missed the target. After a capture attempt (or after the target had left the screen) there was a short pause before the next target presentation began. The experiment used a block design: each of the 7 different target types was presented in a random order in one block, and the full experiment contained 20 blocks, meaning that each participant made 140 capture attempts in total, and each target type was presented 20 times throughout the experiment. We used this number of blocks as it gave a comparable number of target presentations to other studies where learning effects have been seen [33]. Figure 1 shows an example screen shot from this experiment.

Targets were PNG images, created in Image J. Seven different target types were used in this experiment. A luminance matched grey target, high contrast stripe target (spatial frequency of 1.74 cycles/deg) and a ‘background sample’ camouflage target were all luminance matched to the mean background level. The ‘background sample’ target was created by cutting out a random section of the background; each subject saw a target that was cut out from the background exemplar they were presented with. In previous experiments [21], background matching targets had been designed to match a relatively simple, repeating background. In this experiment, we used a more complex, heterogeneous background and matching target to try to replicate the previous findings. A ‘spot’ target and a grey target with white edges were matched in luminance to each other (but not to the previous three targets). As a control, a grey target with luminance matching these two targets was also included. Finally, the 7^th^ target was a uniform white target. The effects of colour were removed from the experiment to allow calibration for luminance and also to simplify the interpretation of the results.

Targets were presented on a ‘heterogeneous’ leafy background, and each participant saw one of four exemplar backgrounds. This was done to ensure that any effects seen were due to the background group and not to a specific image. Heterogeneous backgrounds were chosen as these have been shown to make the prey capture task relatively difficult for subjects [17]. Backgrounds were grey scale digital images of natural substrates, also in PNG format, and each background exemplar was matched for mean luminance. These were sourced from free stock image website www.sxc.hu and Wikipedia.

### Calibration

The display was calibrated for human luminance perception using a Minolta LS-110 luminance meter (Osaka, Japan). Images with grey values ranging from 0–255 on an 8 bit scale were displayed on the screen, and the luminance was measured in lux for each image at four different points on the screen and averaged. The grey value was then plotted against the average luminance to determine the value that would represent an intermediate grey between the black and white markings on a ratio scale, and this value was used in target and background creation. The display refreshed at 70Hz, which would equate to a frame by frame displacement of 0.57 degrees. The flicker of the striped targets was 41.6Hz (based on calculating the time taken for one complete cycle of white and black stripes), which was lower than the refresh rate of the display.

### Subjects

Data from a total of 80 volunteers were used in the analysis (two volunteers were run and not used in the analysis due to technical difficulties with the program). Subjects were drawn from the undergraduate and graduate populations at the University of Cambridge, were naïve to the experimental aims and were only given enough information to be able to play the game. The University of Cambridge's ethical research policies were adhered to and no ethical review was required. Subjects gave consent verbally, and by clicking the “start” button on the touchscreen before the trial commenced. Subjects were free to terminate the trial at any point without explanation, and no sensitive information was collected. Viewing distance was approximately constant at 45 cm, and the experiment was conducted in standard laboratory light conditions throughout the working day (lighting levels did not change with time of day as all windows were covered for the duration of the experiment). All subjects received 10 training target presentations first, where a black target was captured on a white background.

### Statistical analysis

Due to the repeated measures design of the experiment, results were analysed using linear mixed models (LMMs) or generalised linear mixed models (GLMMs) [38,39] using the lme4 package (version 1.1-7) and the lmerTest package (version 2.0-6) in R (version 3.1-0) [40], using target type, background type, trial number and position group (whether the capture attempt was ahead of or behind the midline of the target, as defined by its direction of travel; this factor was included as it greatly improved the model fit, as many more capture attempts were made behind the centre of the target, creating a bimodal distribution) as fixed factors as appropriate. The initial model also contained all possible first order interactions with target type. Subject was included as a random intercept and the specific background exemplar was also included as a random slope. Models were simplified based on their AIC weights and log likelihood to produce a best fit model [38,39]. Analysis was run for each experiment using a hit/miss dependent variable (binomial error structure), and also for a reaction time measure (log normal error structure). We calculated the overall main effects of the models using the Anova function from the car package (version 2.0-20) and then analysed the effects of individual pattern types using planned contrast comparisons [41]. The high contrast striped target was taken as the reference against which all other targets were compared.

## Results

**Figure 2:**
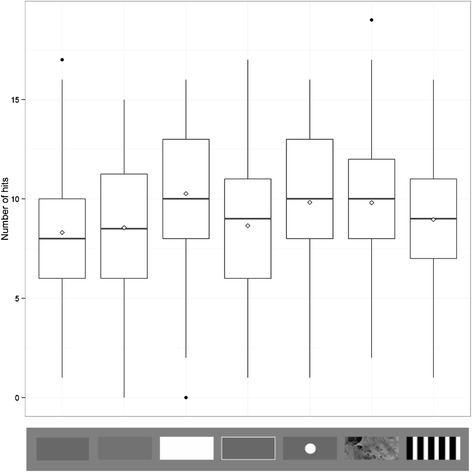
**Distribution of the number of hits for each target type across all subjects and trials.** Trial types from left to right are average background luminance matching grey, lighter grey, white, white edged grey, ‘spot’, background matching camouflage and high contrast stripe. Whiskers encompass 1.5 × the interquartile range, and points beyond this are plotted as outliers (black circles). Means are represented by white diamonds.

All main effects in the simplified hits model were significant (target type: χ^2^ = 47.328, p <0.001, position group: χ^2^ = 98.199, p <0.001, trial number χ^2^ = 65.744, p < 0.001). We then compared the hit rate (hits as a proportion of the total number of attempts) of the high contrast striped target with all the other target types (see Table 2 for full statistical results). When considering the capture rate of these stimuli, there was no significant difference between the striped target and either of the grey targets (Z = −1.520, p = 0.128 for the background average luminance matched grey, and Z = −0.702, p = 0.483 for the lighter grey; Figure 2). There was also no difference between the striped target and the white edged grey target (Z = −0.598, p = 0.550). However, there was a significant difference between the striped target and the white target (Z = 3.660, p < 0.001), the ‘spot’ target (Z = 2.729, p = 0.006) and the background matching camouflage target (Z = 2.126, p = 0.034), with these three targets all being easier to capture than the striped target. There was also a significant effect of position group, with significantly more successful capture attempts made in front of the target centre than behind (Z = 9.910, p < 0.001), and trial number, with participants improving throughout the experiment (Z = 8.108, p < 0.001). During model simplification, the interaction of trial number with target type dropped out of the model, suggesting that learning rates were not different for different targets. Similarly the interaction of position group with target type was not significant and was dropped.

**Table 2:** **Table to show the full statistical results for the hit rate measure**

**Factor**	**Estimate**	**Std. error**	**Z value**	**p value**
Stripe vs. luminance match grey	−0.11405	0.07503	−1.520	0.128
Stripe vs. lighter grey	−0.05262	0.07497	−0.702	0.483
Stripe vs. white	0.27288	0.07455	3.660	<0.001
Stripe vs. white edged grey	−0.04470	0.07476	−0.598	0.550
Stripe vs. ‘spot’	0.20384	0.07470	2.729	0.006
Stripe vs. background match	0.15891	0.07475	2.126	0.034
Position group	0.58129	0.05866	9.910	<0.001
Trial number	0.16294	0.02010	8.108	<0.001

These results were obtained using a generalised linear mixed model. The first six rows detail the planned comparisons of the target type, while the final two rows show the effects of the other factors included in the model.

**Figure 3:**
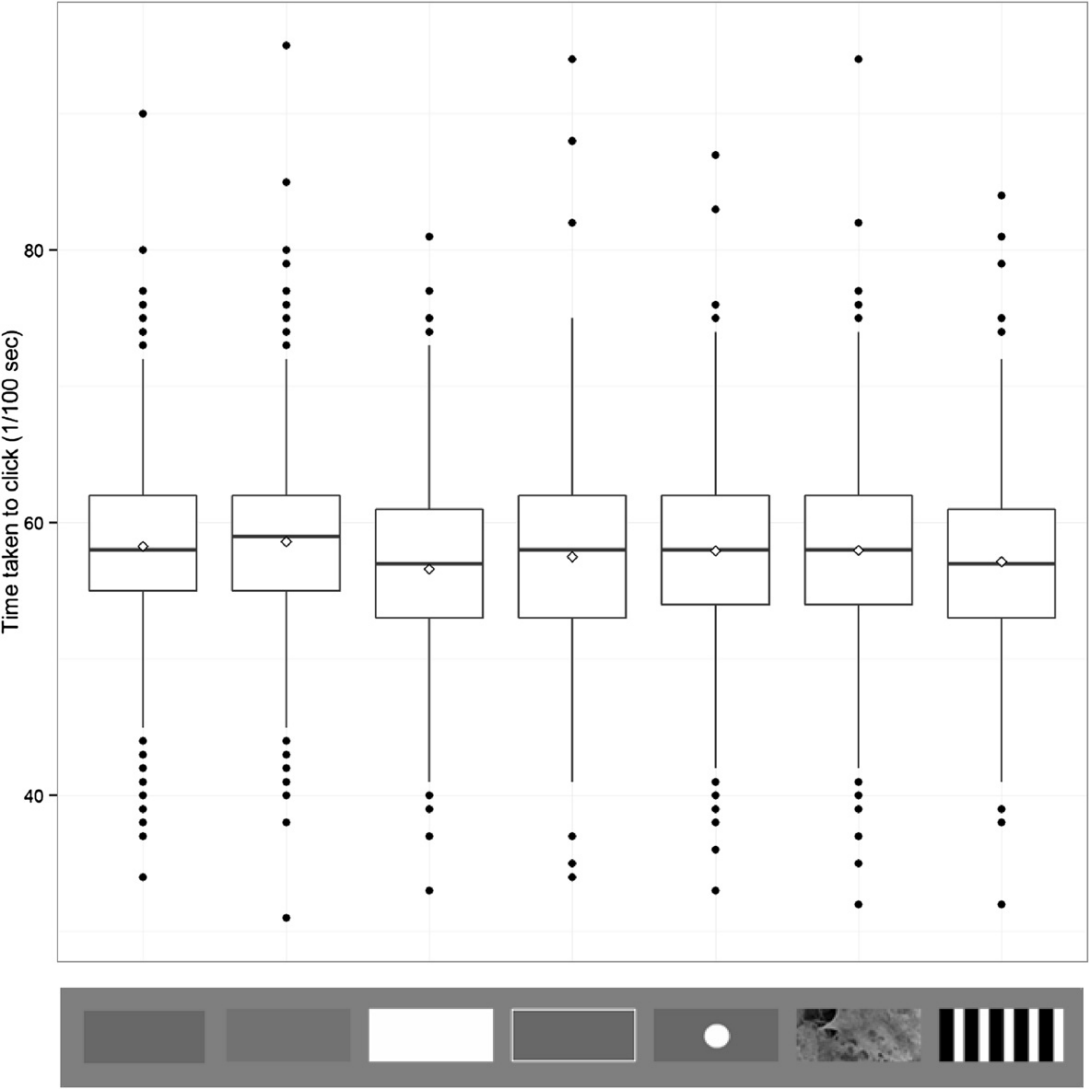
**Distribution of time taken to hit for each target type across all subjects and trials.** Trial types from left to right are average background luminance matching grey, lighter grey, white, white edged grey, ‘spot’, background matching camouflage and high contrast stripe. Whiskers encompass 1.5 x the interquartile range, and points beyond this are plotted as outliers (black circles). Means are represented by white diamonds.

We also used a capture attempt time measure to analyse these data, again finding that all the main effects in our simplified model were significant (target type: χ^2^ = 182.86, p <0.001, position group: χ^2^ = 410.34, p <0.001, trial number χ^2^ = 493.77, p < 0.001). We then compared the high contrast striped target to all other target types (see Table 3 for full results). Participants were slower to make capture attempts to both grey stimuli compared to the striped target (t = 6.289, p < 0.001 for the background average luminance matched grey, and t = 8.154, p < 0.001 for the lighter grey; Figure 3). Participants were also slower to make capture attempts for the ‘spot’ target (t = 4.931, p < 0.001) and for the background matching camouflage target (t = 5.847, p < 0.001). However, there was no significant difference in reaction time between the striped target and the white edged grey target (t = 1.566, p = 0.117). Finally, participants made faster capture attempts to the white target compared to the striped target (t = −2.695, p = 0.007). Again, there was a significant effect of trial number, with participants increasing in speed throughout the experiment (t = 22.221, p < 0.001), and of position group, with significantly quicker capture attempts being made in front of the centre of the target than behind (t = −20.257, p < 0.001). During model simplification, the interaction of trial number with target type dropped out of the model, suggesting that people did not differentially change their capture strategy with different targets. The interaction of position group with target type was also not significant and was dropped.

**Table 3:** **Table to show the full statistical results for the capture time measure**

**Factor**	**Estimate**	**Std. error**	**t value**	**p value**
Stripe vs. luminance match grey	0.01942	0.003087	6.289	<0.001
Stripe vs. lighter grey	0.02518	0.003089	8.154	<0.001
Stripe vs. white	−0.008295	0.003079	−2.695	0.007
Stripe vs. white edged grey	0.004827	0.003083	1.566	0.117
Stripe vs. ‘spot’	0.01522	0.003087	4.931	<0.001
Stripe vs. background match	0.01805	0.003087	5.847	<0.001
Position group	−0.04877	0.002408	−20.257	<0.001
Trial number	0.0184	0.0008255	22.221	<0.001

These results were obtained using a linear mixed model. The first six rows detail the planned comparisons of the target type, while the final two rows show the effects of the other factors included in the model.

## Discussion

The aim of this experiment was to elucidate the relative difficulty of capture of different types of patterned targets. We found that high contrast striped targets were relatively difficult, as in previous experiments, but that this benefit was not unique; several other target types, including uniform grey targets and a white edged grey target were similarly difficult to capture. However, white, ‘spot’ and background sample targets were easier to capture than the high contrast striped target. These findings confirm those shown previously [17,21] and extend them to show that a range of different pattern types can influence capture success. We also found differences in capture attempt time among targets, with participants making faster responses to the white targets and slower responses to the other targets, in comparison to the striped targets. However, we found no differences in learning rates across trials for different targets.

The fact that the striped target was relatively difficult to capture is in agreement with several other recent studies [17,21]. However, success rates for the striped target did not significantly differ from the grey targets. Previous studies have also shown that when stationary, striped patterning is much easier to find than uniform grey patterning in a detection task [21]. Other studies have also found that low contrast targets are similarly effective in preventing capture [17], and that animals prefer to use low contrast patterning when in motion [20]. This suggest that all other things being equal, the uniform luminance matched grey patterning may be effective in preventing capture, as well as having some benefit in preventing detection.

It has previously been hypothesised that high contrast patterns such as stripes may help to provide camouflage when in motion through ‘flicker fusion’, where the rapid movement of the animal blurs the striped pattern causing it to appear uniformly camouflaged [42]. The results from this experiment suggest generally that if an animal moves fast enough to cause flicker fusion, the effect could cause it to become more difficult to capture (as uniform grey targets are relatively difficult to catch) but that if they don’t, stripes may also provide effective motion camouflage. As flicker fusion would depend on many parameters, including the visual acuity of the predator, the speed of the prey, the width of the stripes and the range of the viewer, it would seem likely that this effect would not occur in all predator–prey encounters, and thus the fact that the stripes themselves can also cause perceptual illusions when in motion could be beneficial.

We found that the white target was easier for participants to catch than the striped target, in agreement with some recent studies [17,21], and subjects also made the fastest responses to these targets, suggesting they were confident about making accurate capture attempts [35-37]. Capture attempts were also made relatively quickly to the striped targets, which could suggest that there was a dissociation between perception and action in this task; perceptually participants felt confident about their judgements, but were actually relatively inaccurate in their actions. The speed of response and the confidence felt by subjects could reflect the detectability of the targets, as striped targets have been shown to be easy to detect when stationary [21].

Interestingly, some recent research has shown the opposite effect to that seen in this study, with striped objects being hit more often than uniformly coloured objects [19]. In that earlier study, subjects attempted to hit target objects that were moving from left to right using a cursor controlled by a joystick. On each trial, the target disappeared before the subject made their capture attempt, and so participants had to predict where they thought the target would have reached on its trajectory when making their capture attempt. There are several differences in design of this study that could explain the differences in results seen. Firstly, the unicoloured object in their experiment was black, in comparison to the white target used in the current experiment. While both of these targets would have similar contrasts on a mid-grey background and thus might be expected to give similar results, it is possible that their different luminance levels differentially affect capture success. Secondly, the target trajectory was always the same across trials in the study by Von Helversen and colleagues, and thus was highly predictable, whereas in the current study, there was more variability in target trajectory, with targets being presented on one of 32 trajectories randomly, ensuring that the participants could not predict where it would be travelling on each trial. It would be interesting in future research to know if these differences could explain the variability seen in results.

Striped targets were also significantly more difficult to capture than both the spot and background sampled targets. The latter result is in agreement with previous research [21], and suggests this effect may occur using a range of different backgrounds. One thing that these targets have in common is that they both have ‘features’ in the body of the target, which may have acted as a tracking cue for motion direction and speed [23-27], allowing participants to make more accurate capture attempts. Both of these target types had significantly slower capture attempt times than the striped target, perhaps indicating that participants actively tried to track them, as it is thought that extracting and locating features may take longer than other motion extraction methods [43]. Interestingly, the white edged grey target was relatively difficult to capture, being caught at a statistically similar rate to the striped target, and capture attempts were also made at a similar rate to the striped target. This could suggest that the edges of the target are treated as relatively unimportant, which is surprising as the corners and line ends should also provide ‘feature’ information for tracking. Visual attention is thought to be important in feature tracking [44,45], and therefore it could be that visual attention plays a role in determining which features are used in motion integration. For example, if participants focus their attention on the centre of the target in their attempts to capture it, they may not use the information from the edges.

During statistical modelling, we tested for the effect of differential learning of target types, but did not find any significant effects. While participants tended to improve with increasing exposure to a particular type of target, the rates of improvement were not significantly different for different target types. This contrasts with recent work with stationary camouflage, which suggests that some types of camouflage patterning are learnt more rapidly than others [33], and suggests that differences in learning rate cannot account for the differences in pattern capture success shown.

It is of course important to note that other factors may affect the relative advantages of these different types of patterning, and that these results may not represent the natural situation in several ways. It is known that some prey animals move unpredictably and erratically in their escape behaviours in what is known as protean or demiatic movement [46], and it has been shown that this behaviour might affect capture success [16]. In addition, some animals commonly travel in groups, and increased group size has been shown to decrease capture success via the confusion effect [47,48]. It is possible that the patterning on each animal could interact in a non-linear fashion with the confusion effect or protean behaviours to create a stronger effect. In accordance with this, modelling potential motion detection mechanisms in zebra herds has shown that the motion signals created are varied, perhaps leading to both misperceptions in motion perception and difficulties in perceptually isolating individual zebra for predators or parasites [16]. Patterning may also have evolved for multiple purposes, including aposematism and mate choice, which may need to be considered in assessing the relative costs and benefits of different types of patterning. For example, in zebra, it has been suggested that stripes may be unattractive to insect pests [49-51] and in some snake species, zigzag markings may have an aposematic function [52]. In some species, conspicuous stripes may pose relatively little cost, as they offer similar camouflage benefits to the uniform grey coloration when in motion, while still allowing the range of other communicative signals that dazzle signals can be used for. Finally, different animals have different visual systems, and prey patterning may therefore be specialised for the parameters of the particular predators they encounter. An important avenue for further research would be to test the current findings in other animal systems as well as human models.

## Conclusions

In conclusion, we have shown clear differences in capture success for different types of target patterning in this study. Our results suggest that there is not a unique benefit for putative dazzle-style patterning, as some uniform grey low contrast targets are also difficult to capture, in agreement with previous results. However, striped patterning does seem to have properties that are conducive to preventing capture, compared to some other target types. Despite subjects responding relatively quickly to striped targets, suggesting that they find them easy to detect or that they expect them to be easy to capture, these targets are actually more difficult to catch than several other types of target patterning. Interestingly, one common feature of targets that appear to be relatively easy to catch is the presence of easily trackable features in the centre of the target. A future challenge is therefore to understand why the highly visible stripes do not seem to be able to be used for accurate tracking in the same manner.

## References

[1] Stevens M, Merilaita S (2009). Animal camouflage: current issues and new perspectives. Philos Trans R Soc B Biol Sci.

[2] Stevens M (2007). Predator perception and the interrelation between different forms of protective coloration. Proc R Soc B Biol Sci.

[3] Cuthill IC, Stevens M, Sheppard J, Maddocks T, Párraga CA, Troscianko TS (2005). Disruptive coloration and background pattern matching. Nature.

[4] Stevens M, Marshall KLA, Troscianko J, Finlay S, Burnand D, Chadwick SL (2013). Revealed by conspicuousness: distractive markings reduce camouflage. Behav Ecol.

[5] Rowland HM, Speed MP, Ruxton GD, Edmunds M, Stevens M, Harvey IF (2007). Countershading enhances cryptic protection: an experiment with wild birds and artificial prey. Anim Behav.

[6] Rowland HM, Cuthill IC, Harvey IF, Speed MP, Ruxton GD (2008). Can’t tell the caterpillars from the trees: countershading enhances survival in a woodland. Proc R Soc B Biol Sci.

[7] Fraser S, Callahan A, Klassen D, Sherratt TN (2007). Empirical tests of the role of disruptive coloration in reducing detectability. Proc R Soc B Biol Sci.

[8] Schaefer HM, Stobbe N (2006). Disruptive coloration provides camouflage independent of background matching. Proc R Soc B Biol Sci.

[9] Webster RJ, Hassall C, Herdman CM, Godin J-GJ, Sherratt TN (2013). Disruptive camouflage impairs object recognition. Biol Lett.

[10] Behrens RR (1999). The role of artists in ship camouflage during World War I. Leonardo.

[11] Forbes P (2011). Dazzled and Deceived: Mimicry and Camouflage.

[12] Shine R, Madsen T (1994). Sexual dischromatism in snakes of the genus vipera: a review and a new evolutionary hypothesis. J Herpetol.

[13] Brodie ED (1993). Differential avoidance of coral snake banded patterns by free-ranging avian predators in Costa Rica. Evolution.

[14] Jackson JF, Ingram W, Campbell HW (1976). The dorsal pigmentation pattern of snakes as an antipredator strategy: a multivariate approach. Am Nat.

[15] Allen WL, Baddeley R, Scott-Samuel NE, Cuthill IC (2013). The evolution and function of pattern diversity in snakes. Behav Ecol.

[16] How MJ, Zanker JM (2014). Motion camouflage induced by zebra stripes. Zool Jena Ger.

[17] Stevens M, Yule DH, Ruxton GD (2008). Dazzle coloration and prey movement. Proc R Soc B Biol Sci.

[18] Scott-Samuel NE, Baddeley R, Palmer CE, Cuthill IC (2011). Dazzle camouflage affects speed perception. PloS One.

[19] Von Helversen B, Schooler LJ, Czienskowski U (2013). Are stripes beneficial? Dazzle camouflage influences perceived speed and hit rates. PloS One.

[20] Zylinski S, Osorio D, Shohet AJ (2009). Cuttlefish camouflage: context-dependent body pattern use during motion. Proc R Soc B Biol Sci.

[21] Stevens M, Searle WT, Seymour JE, Marshall KL, Ruxton GD (2011). Motion dazzle and camouflage as distinct anti-predator defenses. BMC Biol.

[22] Adelson EH, Movshon JA (1982). Phenomenal coherence of moving visual patterns. Nature.

[23] Castet E, Lorenceau J, Shiffrar M, Bonnet C (1993). Perceived speed of moving lines depends on orientation, length, speed and luminance. Vision Res.

[24] Lorenceau J, Shiffrar M (1992). The influence of terminators on motion integration across space. Vision Res.

[25] Lorenceau J, Shiffrar M, Wells N, Castet E (1993). Different motion sensitive units are involved in recovering the direction of moving lines. Vision Res.

[26] Nakayama K, Silverman GH (1988). The aperture problem–I. Perception of nonrigidity and motion direction in translating sinusoidal lines. Vision Res.

[27] Nakayama K, Silverman GH (1988). The aperture problem–II. Spatial integration of velocity information along contours. Vision Res.

[28] Shimojo S, Silverman GH, Nakayama K (1989). Occlusion and the solution to the aperture problem for motion. Vision Res.

[29] Hubel DH, Wiesel TN (1968). Receptive fields and functional architecture of monkey striate cortex. J Physiol.

[30] Marr D, Hildreth E (1980). Theory of edge detection. Proc R Soc Lond - Biol Sci.

[31] Hildreth EC (1984). The computation of the velocity field. Proc R Soc Lond - Biol Sci.

[32] Marr D, Ullman S (1981). Directional selectivity and its use in early visual processing. Proc R Soc Lond Ser B Contain Pap Biol Character R Soc G B.

[33] Troscianko J, Lown AE, Hughes AE, Stevens M (2013). Defeating crypsis: detection and learning of camouflage strategies. PLoS One.

[34] Santer RD (2013). Motion dazzle: a locust’s eye view. Biol Lett.

[35] Kellogg WN (1931). The time of judgment in psychometric measures. Am J Psychol.

[36] Henmon V a C (1911). The relation of the time of a judgment to its accuracy. Psychol Rev.

[37] Audley RJ (1960). A stochastic model for individual choice behavior. Psychol Rev.

[38] Crawley MJ (2005). Statistics: An Introduction Using R.

[39] Zuur AF, Ieno EN, Walker NJ, Saveliev AA, Smith GM (2009). Mixed Effects Models and Extensions in Ecology with R.

[40] Ihaka R, Gentleman R (1996). R: A Language for data analysis and graphics. J Comput Graph Stat.

[41] Ruxton GD, Beauchamp G (2008). Time for some a priori thinking about post hoc testing. Behav Ecol.

[42] Pough FH (1976). Multiple cryptic effects of crossbanded and ringed patterns of snakes. Copeia.

[43] Derrington AM, Allen HA, Delicato LS (2004). Visual mechanisms of motion analysis and motion perception. Annu Rev Psychol.

[44] Allen HA, Derrington AM (2000). Slow discrimination of contrast-defined expansion patterns. Vision Res.

[45] Ashida H, Seiffert AE, Osaka N (2001). Inefficient visual search for second-order motion. J Opt Soc Am A Opt Image Sci Vis.

[46] Humphries DA, Driver PM (1967). Erratic display as a device against predators. Science.

[47] Tosh CR, Jackson AL, Ruxton GD (2006). The confusion effect in predatory neural networks. Am Nat.

[48] Jeschke JM, Tollrian R (2007). Prey swarming: which predators become confused and why?. Anim Behav.

[49] Ruxton GD (2002). The possible fitness benefits of striped coat coloration for zebra. Mammal Rev.

[50] Gibson G (1992). Do tsetse-flies see zebras -a field-study of the visual response of tsetse to striped targets. Physiol Entomol.

[51] Caro T, Izzo A, Reiner RC, Walker H, Stankowich T (2014). The function of zebra stripes. Nat Commun.

[52] Valkonen J, Niskanen M, Björklund M, Mappes J (2011). Disruption or aposematism? Significance of dorsal zigzag pattern of European vipers. Evol Ecol.

